# Genetic diversity and amino acid sequence polymorphism in *Helicobacter pylori *CagL hypervariable motif and its association with virulence markers and gastroduodenal diseases

**DOI:** 10.1002/cam4.1941

**Published:** 2019-03-14

**Authors:** Abbas Yadegar, Ashraf Mohabati Mobarez, Mohammad Reza Zali

**Affiliations:** ^1^ Foodborne and Waterborne Diseases Research Center, Research Institute for Gastroenterology and Liver Diseases Shahid Beheshti University of Medical Sciences Tehran Iran; ^2^ Department of Bacteriology, Faculty of Medical Sciences Tarbiat Modares University Tehran Iran; ^3^ Gastroenterology and Liver Diseases Research Center, Research Institute for Gastroenterology and Liver Diseases Shahid Beheshti University of Medical Sciences Tehran Iran

**Keywords:** *cagL*, CagLHM, disease outcome, diversity, *Helicobacter pylori*, Iran, virulence factors

## Abstract

Genetic variability in *cagL* gene especially within the *Helicobacter pylori* CagL hypervariable motif (CagLHM) may affect the development of gastric cancer. Therefore, this study was conducted to investigate the association of CagL diversity with clinical outcomes and with *H pylori* virulence markers. A total of 126 patients with different gastric diseases including non‐ulcer dyspepsia (NUD), peptic ulcer disease (PUD), gastric erosion (GE), and gastric cancer (GC) were enrolled. *H pylori* was cultured from gastric biopsies, and the isolates were screened for the presence of *cagL*, *cagA*, *vacA*, *babA2*, *sabA*, and *cag*PAI integrity by PCR. The amino acid polymorphisms of *cagL* were analyzed using DNA sequencing. We isolated 61 (48.4%) *H pylori* strains from 36 NUD, eight PUD, 12 GE, and five GC patients. Almost all isolates were *cagL* positive (97%), and their RGD, RHS, and SKIIVK motifs were highly conserved. Among 10 CagLHM variants identified, NEIGQ and NKIGQ were detected as the most prevalent sequences. Interestingly, a significant association was found between the presence of NKMGK and PUD (*P* = 0.002). Notably, the NEIGQ isolates with multiple C‐type EPIYA repeat that carried intact *cag*PAI correlated with disease risk for PUD, GE, and GC (*P* = 0.021). In conclusion, we identified novel variants of *H pylori* CagLHM sequences in Iranian population such as NKMGK, which was associated with disease risk for PUD. Further studies using a large number of strains are required to better clarify the function of certain CagLHM motifs in gastric carcinogenesis and disease outcome.

## 1 INTRODUCTION

The spiral‐shaped microaerophilic bacterium, *Helicobacter pylori*, is classified as a group I carcinogen currently regarded as the most common etiologic cause of infection‐related cancers.^1^, ^2^ *H pylori*‐infected individuals have an increased risk of developing gastroduodenal diseases, including chronic gastritis, peptic ulcers, gastric adenocarcinoma, and gastric lymphoma.^3^, ^4^ This highly adapted human gastric pathogen is one of the most genetically diverse bacterial species and displays remarkable genetic variability and microevolution even among closely related strains due to high rate of mutation and recombination events.^5^, ^6^, ^7^, ^8^ Dozens of bacterial factors have been identified to promote the pathogenesis of *H pylori *infections, including cytotoxin‐associated gene A protein (CagA), vacuolating cytotoxin A (VacA), outer inflammatory protein A (OipA), and several putative adherence factors such as the blood‐group antigen‐binding adhesin (BabA) and sialic acid‐binding adhesin (SabA).^9^, ^10^, ^11^, ^12^

A hallmark of the most virulent *H pylori *strains is the presence of an intact *cag* pathogenicity island (*cag*PAI), which is associated with severe gastric pathologies including gastric mucosal inflammation, atrophy, and cancer.^13^, ^14^, ^15^, ^16^ The *cag*PAI is approximately 40 kb long and contains 28‐31 genes encoding a multi‐component bacterial type IV secretion system (T4SS).^17^, ^18^ After bacterial attachment, the T4SS delivers the CagA oncogenic protein and also peptidoglycan into the host gastric epithelial cells.^18^, ^19^ Upon translocation into the host cell, CagA undergoes tyrosine phosphorylation at its carboxy‐terminal Glu‐Pro‐Ile‐Tyr‐Ala (EPIYA) motifs by a variety of cellular kinases. Consequently, translocated CagA interferes with various cell signaling cascades that regulate cell‐cell adhesion, cell proliferation, and elongation and induces host epithelial cell secretion of potent proinflammatory chemokines such as interleukin (IL)‐8.^20^, ^21^

T4SS‐mediated CagA translocation across the host cell membrane depends on a number of bacterial and host cofactors such as CagL and human integrin β_1_‐containing receptors, particularly integrin α_5_β_1_.^18^, ^22^ CagL protein is a pilin‐like component of T4SS encoded by the *cagL* gene (HP0539) and is proposed to be expressed on the surface of *H pylori* in a T4SS‐dependent manner.^17^, ^23^ The arginine‐glycine‐aspartate (RGD) tripeptide motif at residues 76‐78 of CagL and its neighboring surface‐exposed FEANE (Phe‐Glu‐Ala‐Asn‐Glu) motif, referred to as RGD helper sequence (RHS), maximize proposed to be essential for T4SS interaction with integrin receptors for translocation of bacterial effectors into the host cells.^24^ CagL itself can also trigger intracellular signaling pathways by RGD‐dependent binding to integrins and can induce cell proinflammatory responses independently of CagA translocation.^25^, ^26^, ^27^

Recent studies have shown that particular polymorphisms at amino acid residues 58‐62 upstream of the critical RGD motif, referred to as CagL hypervariable motif (CagLHM), may correlate with severe disease progression in a geographically dependent manner.^28^, ^29^ More specifically, CagL amino acid polymorphisms Y58/E59, D58/K59, and N58 may correlate with higher corpus inflammation and integrin α_5_β_1_ expression in the upper stomach, induction of hypochlorhydria vicious cycle, and subsequently with an increase in the risk of gastric carcinogenesis.^30^, ^31^, ^32^, ^33^ Given these findings, and those of our previous study showing a very high prevalence of *cagL* gene among Iranian *H pylori* strains,^34^ here we aimed to characterize the diversity of CagL sequence polymorphisms and investigate whether these polymorphisms associate with clinical outcomes in patients with different gastroduodenal diseases. Associations between CagL sequence polymorphisms and different *H pylori* virulence genotypes were also investigated.

## 2 MATERIALS AND METHODS

### 2.1 Patients and gastric biopsies

We enrolled 126 patients suffering from different gastroduodenal diseases who underwent standard upper gastrointestinal endoscopy at Research Institute for Gastroenterology and Liver Diseases, Tehran, Iran, from January 2011 to May 2012. Three gastric biopsies were taken from the antrum of the stomach of each patient for *H pylori* culture and histopathological examination. The biopsy specimen for isolation of *H pylori* strains were immediately kept in transport medium containing thioglycolate with 1.3 g/L agar (Merck, Germany) and 3% yeast extract (Oxoid Ltd., Basingstoke, UK). Written informed consent was obtained from all patients under a protocol approved by the Ethical Review Committee of the Gastroenterology and Liver Diseases Research Institute at Shahid Beheshti University of Medical Sciences.

### 2.2 *H* *pylori* growth conditions and identification

The fresh gastric biopsy samples were completely dissected, homogenized, and cultured on the surface of Brucella agar plates (Merck) supplemented with 7% (v/v) horse blood, 10% fetal calf serum (FCS), *Campylobacter*‐selective supplement (vancomycin 2.0 mg/L, polymyxin 0.05 mg/L, trimethoprim 1.0 mg/L), and amphotericin B (2.5 mg/L). The cultured plates were incubated at 37°C under microaerobic conditions (5% O_2_, 10% CO_2_, and 85% N_2_) in a CO_2_ incubator for 3‐7 days. Bacterial growth was identified as *H pylori *by colony morphology and Gram stain, as well as positive reactions for oxidase, catalase, and urease, and subsequently by species‐specific PCR assays as previously described.^34^, ^35^ Pure cultures from each strain were harvested and stored at −80°C in 0.5 mL of brain heart infusion (BHI) medium (Merck) containing 15% glycerol plus 20% FCS until further experiments.

### 2.3 DNA extraction and virulence genotyping

Genomic DNA was extracted from sweeps of primary *H pylori* colonies using the QIAamp DNA Mini Kit (QIAGEN GmbH, Hilden, Germany) as per the manufacturer's instructions. Purified DNA samples were stored at −20°C until required for PCR analyses. The quality and specificity of DNA samples were confirmed using a NanoDrop ND‐1000 spectrophotometer (NanoDrop Technologies, Wilmington, DE, USA). *H pylori* 16S rRNA‐ and *glmM*‐specific PCRs, which produced the expected amplicon sizes of 764 and 296 bp, were also performed for all strains. Virulence factor genotyping of each *H pylori* isolate was performed by *cagL*‐, *cagA*‐, *babA2*‐, *sabA*‐, and *vacA*‐specific PCRs using previously published primer pairs and PCR conditions.^34^ *H pylori* J99 (CCUG 47164) and a no‐template reaction served as positive and negative controls in each PCR experiment, respectively.

### 2.4 Determination of CagA EPIYA motifs and *cag*PAI integrity

For determining the CagA EPIYA motifs, the *cagA* gene 3′ variable region was amplified using specific primers 5′ TCCGTTAAAGATGTGATCATCAATC 3′ (cag3′F) and 5′ AGATTTTTGGA AACCACCTTTTG 3′ (cag3′R), as previously described.^16^ *cag*PAI integrity was investigated by multiple PCRs using eleven sets of specific oligonucleotide primers spanning the *cag*PAI locus as per our previously described scheme.^16^, ^36^ The *cag*PAI was defined as intact/complete when all the selected gene segments were present. Partially deleted *cag*PAI was defined as where some, but not all, of the *cag*PAI gene segments were present. Complete absence of the *cag*PAI was confirmed by a simple PCR amplification using Luni1 and R5280 primers yielding a 550 bp empty *cag*PAI site amplicon.^37^

### 2.5 Sequencing of *cagL* genes

For DNA sequencing of *cagL* genes, 25 µL PCRs using specific primers CagL‐B4 (5′ GCAGAATTCATAACAAGCGGCTTAAAG 3′) and CagL‐B5 (5′ ATTAGAATTCATAGCCTATCGTCTCAG 3′) generated 695 bp PCR amplicons. The PCR products were purified using the Silica Bead DNA Gel Extraction Kit (Thermo Scientific, Fermentas, USA). The partial nucleotide sequences of *cagL* genes from 46 strains characterized in this study were deposited in the GenBank database; accession numbers are shown in Table 1.

**Table 1:** Distribution of 46 *H pylori cagL*‐positive isolates in relation to clinical status and demographic data of the respective patients

No.	Strain	GenBank Accession No.^a^	Clinical status	Gender (F/M)	Age (years)	Ethnicity
1	HpOC179	KC609279.1	GC	F	63	Fars
2	HpNOC293	KC609280.1	GC	F	52	Turk
3	HpNOC560	KC609281.1	GC	F	54	Fars
4	HpOC485	KC609282.1	NUD	M	54	Fars
5	HpOC494	KC609283.1	NUD	F	42	Fars
6	HpOC557	KC609284.1	PUD	F	50	Fars
7	HpOC571	KC609285.1	NUD	F	49	Fars
8	HpOC573	KC609286.1	NUD	F	36	Turk
9	HpOC576	KC621877.1	NUD	F	42	Fars
10	HpOC606	KC621878.1	GE	M	60	Fars
11	HpOC639	KC621879.1	GE	M	25	Lur
12	HpOC656	KC621880.1	GE	M	41	Turk
13	HpOC658	KC621881.1	NUD	F	33	Fars
14	HpOC723	KC621882.1	NUD	M	47	Turk
15	HpOC728	KC621883.1	NUD	F	23	Turk
16	HpOC734	KC621884.1	NUD	M	50	Fars
17	HpOC743	KC621885.1	NUD	M	60	Turk
18	HpOC751	KC621886.1	NUD	F	44	Fars
19	HpOC770	KC621887.1	NUD	F	73	Fars
20	HpOC775	KC621888.1	GE	F	39	Fars
21	HpOC785	KC621889.1	NUD	M	60	Turk
22	HpOC790	KC621890.1	NUD	M	26	Fars
23	HpOC793	KC621891.1	NUD	F	41	Fars
24	HpOC796	KC621892.1	GE	F	51	Fars
25	HpOC797	KC621893.1	NUD	F	28	Fars
26	HpOC803	KC621894.1	NUD	M	52	Fars
27	HpOC805	KC621895.1	NUD	F	48	Fars
28	HpOC808	KC621896.1	NUD	F	65	Lur
29	HpOC810	KC621897.1	NUD	F	53	Lur
30	HpOC814	KC621898.1	PUD	F	25	Lur
31	HpOC815	KC621899.1	NUD	F	34	Fars
32	HpOC816	KC621900.1	NUD	M	14	Fars
33	HpOC819	KC621901.1	GE	F	32	Turk
34	HpOC824	KC621902.1	PUD	F	43	Turk
35	HpOC852	KC621903.1	NUD	M	45	Fars
36	HpOC854	KC621904.1	NUD	F	71	Fars
37	HpOC897	KC621905.1	PUD	F	60	Fars
38	HpOC912	KC621906.1	PUD	F	64	Fars
39	HpOC913	KC621907.1	PUD	M	40	Fars
40	HpOC937	KC621908.1	NUD	F	48	Fars
41	HpOC939	KC621909.1	PUD	M	54	Turk
42	HpOC975	KC621910.1	GE	F	31	Fars
43	HpOC996	KC621911.1	GE	F	52	Fars
44	HpOC1021	KC621912.1	GE	F	50	Fars
45	HpOC1028	KC621913.1	NUD	F	27	Turk
46	HpOC1031	KC621914.1	NUD	F	52	Fars

GC, gastric cancer; GE, gastric erosion; NUD, non‐ulcer dyspepsia; PUD, peptic ulcer disease.

a The accession numbers are deposited in GenBank database for *cagL* gene sequences of the *H pylori* strains in this study.

### 2.6 Sequence and phylogenetic analysis

DNA sequences were edited by Chromas Lite version 2.5.1 (Technelysium Pty Ltd, Australia) and BioEdit version 7.2.5 softwares.^38^ CagL peptide sequences were aligned to the sequence of *H pylori* strain P12 (GenBank: ACJ07700.1) as a reference sequence. The single nucleotide polymorphisms and codon usage of the *cagL* sequences were examined using BioEdit version 7.2.5 after in‐frame translation. Neighbor‐joining phylogenetic trees were constructed from both CagL nucleotide and peptide sequences of 46 *H pylori *isolates using Molecular Evolutionary Genetics Analysis version 7.0 (MEGA7).^39^ Additionally, we compared the diversity and frequency of CagLHM sequences in this study with the available global motifs at this location by utilizing the 554 CagL amino acid sequences cited in Supplementary data of a study by Gorrell et al^29^ For clarification, this previously published dataset includes CagL sequences of the 46 *H pylori* isolates characterized in this current study.

### 2.7 Statistical analysis

Data were analyzed using IBM SPSS Statistics for Windows version 21.0 (IBM Corp., Armonk, NY). Significant associations between *cagL* amino acid polymorphisms and *H pylori* virulence genotypes in relationship to gastroduodenal diseases were assessed by Fisher's exact test. All statistical tests were two‐sided, and differences were considered statistically significant when *P* values <0.05.

## 3 RESULTS

### 3.1 Patient characteristics

Among the 126 patients that underwent upper gastrointestinal endoscopy, 61 (48.4%) patients had defined *H pylori* infection based on both positive histology and culture results. Detailed endoscopic diagnoses of these patients have been reported previously.^34^ Briefly, 36 had non‐ulcer dyspepsia (NUD), eight had peptic ulcer disease (PUD), 12 had gastric erosion (GE), and five had gastric cancer (GC). There was no significant difference in the age, gender, and ethnicity between these *H pylori*‐positive patients with different clinical outcomes (*P* > 0.05).

### 3.2 *H pylori *genotyping and *vacA* allelic diversity

Virulence genotypes previously determined for these isolates were *cagL*, *cagA*, *babA2,* and *sabA* carriage by 97% (59/61), 85% (52/61), 97% (59/61), and 84% (51/61) of the strains, respectively, and *vacA* genotypes s1m1 in 29% (18/61), s1m2 in 46% (28/61), and s2m2 in 25% (15/61) of the strains.^34^ No association was identified between virulence genotypes and clinical outcomes.

### 3.3 Diversity of CagA EPIYA motifs and *cag*PAI integrity

All 52 *cagA*‐positive *H pylori* isolates were found to contain the *cagA* 3*'*‐end region expressing distinct EPIYA motifs. Among these isolates, various *cagA* EPIYA types were detected as follows: ABC in 39, ABCC in 7, ABCCC in 1, and multiple EPIYA motifs of different sizes, indicating mixed infections, were detected in five strains. The East Asian CagA containing EPIYA‐D motif was not detected in the examined strains. Further PCR analysis was performed to evaluate the intactness of *cag*PAI locus from the 5′ to the 3′ end in all *H pylori* strains. Accordingly, 70% (43/61) of the isolates carried an intact *cag*PAI, 26% (16/61) carried a partial *cag*PAI and 3% (2/61) completely lacked the *cag*PAI genes. A significant association was found between patients infected with the isolates carrying intact *cag*PAI plus multiple C‐type EPIYA repeats and more severe clinical outcomes including PUD, GE, and GC (*P* = 0.013). In addition, patients ≥50 years infected with the isolates carrying intact *cag*PAI had a significant disease risk for PUD, GE, and GC (*P* = 0.038) than NUD.

### 3.4 CagL sequence diversity in disease outcomes

Although the expected 695 bp *cagL* amplicon was obtained from all 59 *cagL*‐genopositive *H pylori* clinical isolates, direct sequencing produced only 46 sequences of sufficient quality for *cagL* polymorphism analysis, including 27 NUD, 7 PUD, nine GE, and three GC isolates. Details of these strains and corresponding patients’ details, including clinical status, gender, age, and ethnicity, are presented in Table 1. The *cagL* sequences were manually edited and trimmed, and aligned with sequences available in the NCBI GenBank database. The *cagL* nucleotide sequences of our strains, which showed >97% homology with the *cagL* gene of the reference *H pylori* strain P12, were translated to amino acid sequences using BioEdit software.

**Figure 1:**
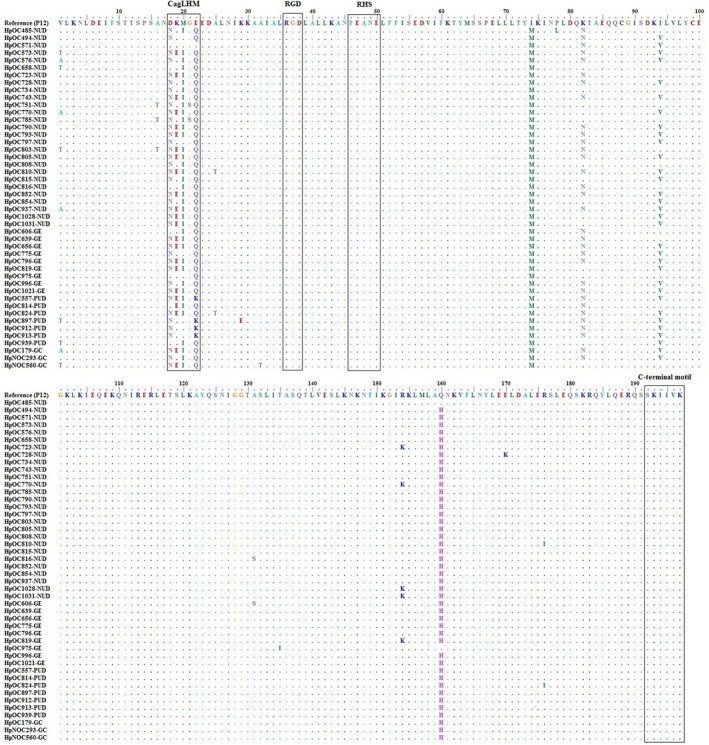
Partial amino acid sequence alignment of CagL from 46 *H pylori* clinical strains from patients with different gastric diseases. The CagL amino acid sequence of the *H pylori* reference strain P12 is shown on the top line. The clinical strains included 27 from non‐ulcer dyspepsia (NUD), seven from peptic ulcer disease (PUD), nine from gastric erosion (GE), and three from gastric cancer (GC) patients. Sequences of CagL hypervariable motif (CagLHM), conserved arginine‐glycine‐aspartate motifs (RGD), RGD helper sequence (RHS) motifs comprising the FEANE (Phe‐Glu‐Ala‐Asn‐Glu) sequence, and highly conserved C‐terminal hexapeptide motifs consisting of the SKIIVK (Ser‐Lys‐Ile‐Ile‐Val‐Lys) sequence are surrounded by boxes

The frequency of synonymous and non‐synonymous *cagL* nucleotide polymorphism for 46 *H pylori* strains are presented in Table 2 and Figure 1. The most variable codon usage was observed at residues 41, 62, 122, and 171 with amino acid polymorphisms including V/T/A, E/Q/K, K/N, and A/S, respectively. The frequency of V41 amino acid sequence polymorphism in patients with NUD (77.8%) and PUD (71.4%) was higher compared to GC patients in which V/T/A substitutions were equally distributed. However, the presence of V41 was found in all patients with GE (100%). The rate of D58 (7, 15.2%) amino acid substitution was significantly lower in comparison with N58 (39, 84.8%) among all patients with different gastric diseases. However, the occurrence rate of K59 vs E59 substitution was found to be approximately equal in different gastric diseases apart from GC patients with higher rate of E59. Moreover, all isolates (100%) with K62 substitution were PUD cases. The presence of N58 mostly accompanied E59 than K59 (22 vs 17 residue combinations), while the presence of D58 mostly accompanied K59 than E59 (6 vs 1 residue combinations). The combined residues N58E59 had higher rates in NUD (13/27, 48.1%), GE (5/9, 55.5%), and GC (2/3, 66.7%) patients compared to patients with PUD (2/7, 28.6%). In contrast, the N58K59 amino acid combination was more frequently found among PUD (3/7, 42.8%) patients than other disease outcomes. However, no significant associations were observed between these combined residue polymorphisms and clinical outcomes (*P* > 0.05).

**Table 2:** Distribution of amino acid and nucleotide substitutions in CagL of 46 *H pylori* isolates from patients with different gastric diseases

CagL polymorphic residue^a^	Amino acid diversity	Number of disease state‐associated *H pylori* isolates carrying each amino acid polymorphism			
		NUD (*n* = 27)	PUD (*n* = 7)	GE (*n* = 9)	GC (*n* = 3)
41	V/T/A	21:3:3	5:2:0	9:0:0	1:1:1
		GT(G/**A**):**AC**G:G**C**G^b^	GTG:**AC**G	GT(G/**A)**	GTG:**AC**G:G**C**G
56	A/T	24:3	7:0	9:0	3:0
		GCT:**A**CT	GCT	GCT	GCT
58	D/N	3:24	2:5	2:7	0:3
		GAT:**A**AT	GAT:**A**AT	GAT:**A**AT	**A**AT
59	K/E	14:13	4:3	4:5	1:2
		AAA:**G**AA	AAA:**G**AA	AAA:**G**AA	AAA:**G**AA
60	M/I	3:24	3:4	2:7	0:3
		ATG:AT**A**	ATG:AT**A**	ATG:AT**A**	AT**A**
61	G/S	25:2	7:0	9:0	3:0
		GGT:**A**GT	GGT	GGT	GGT
62	E/Q/K	1:26:0	0:3:4	0:9:0	0:3:0
		GAA:**C**AA	**C**AA**:A**AA	**C**AA	**C**AA
65	A/T	26:1	6:1	9:0	3:0
		GCT:**A**CT	GCT:**A**CT	GCT	GCT
69	K/E	27:0	6:1	9:0	3:0
		AAA	AAA:**G**AA	AAA	AAA
72	A/T	27:0	7:0	9:0	2:1
		GCC	GCC	GCC	GCC:**A**CC
114	I/M^c^	0:27	0:7	0:9	0:3
		AT**G**	AT**G**	AT**G**	AT**G**
118	P/L	26:1	7:0	9:0	3:0
		CCC:C**T**C	CCC	CCC	CCC
122	K/N	10:17	1:6	2:7	1:2
		AAG:AA(**C**/**T)**	AAG:AA(**C**/**T)**	AA(G/**A**):AA(**C**/**T)**	AAG:AA(**C**/**T)**
134	I/V	9:18	1:6	3:6	1:2
		ATT:**G**TT	ATT:**G**TT	ATT:**G**TT	ATT:**G**TT
171	A/S	26:1	7:0	8:1	3:0
		GCT:**T**CT	GCT	GCT:**A**CT	GCT
175	T/I	27:0	7:0	8:1	3:0
		ACT	ACT	ACT:A**T**T	ACT
194	R/K	23:4	7:0	8:1	3:0
		AGA:A**A**A	AGA	AGA:A**A**A	AGA
200	Q/H	1:26	0:7	1:8	0:3
		CAA:CA**C**	CA**C**	CAA:CA**C**	CA**C**
210	E/K	26:1	7:0	9:0	3:0
		GAG:**A**AG	GAG	GAG	GAG
216	R/I	26:1	6:1	9:0	3:0
		AGA:A**T**A	AGA:A**T**A	AGA	AGA

GC, gastric cancer; GE, gastric erosion; NUD, non‐ulcer dyspepsia; PUD, peptic ulcer disease.

a Positions of amino acid residues correspond to the *H pylori* P12 reference strain.

b Codon usage in italics; bolded nucleotides represent nucleotide polymorphisms.

c Denotes that all of our strains had M (ATG) residue in this position in comparison to *H pylori* P12 that had I (ATA).

### 3.5 High sequence conservation of RGD, RHS, and C‐terminal motifs of CagL

**Figure 2:**
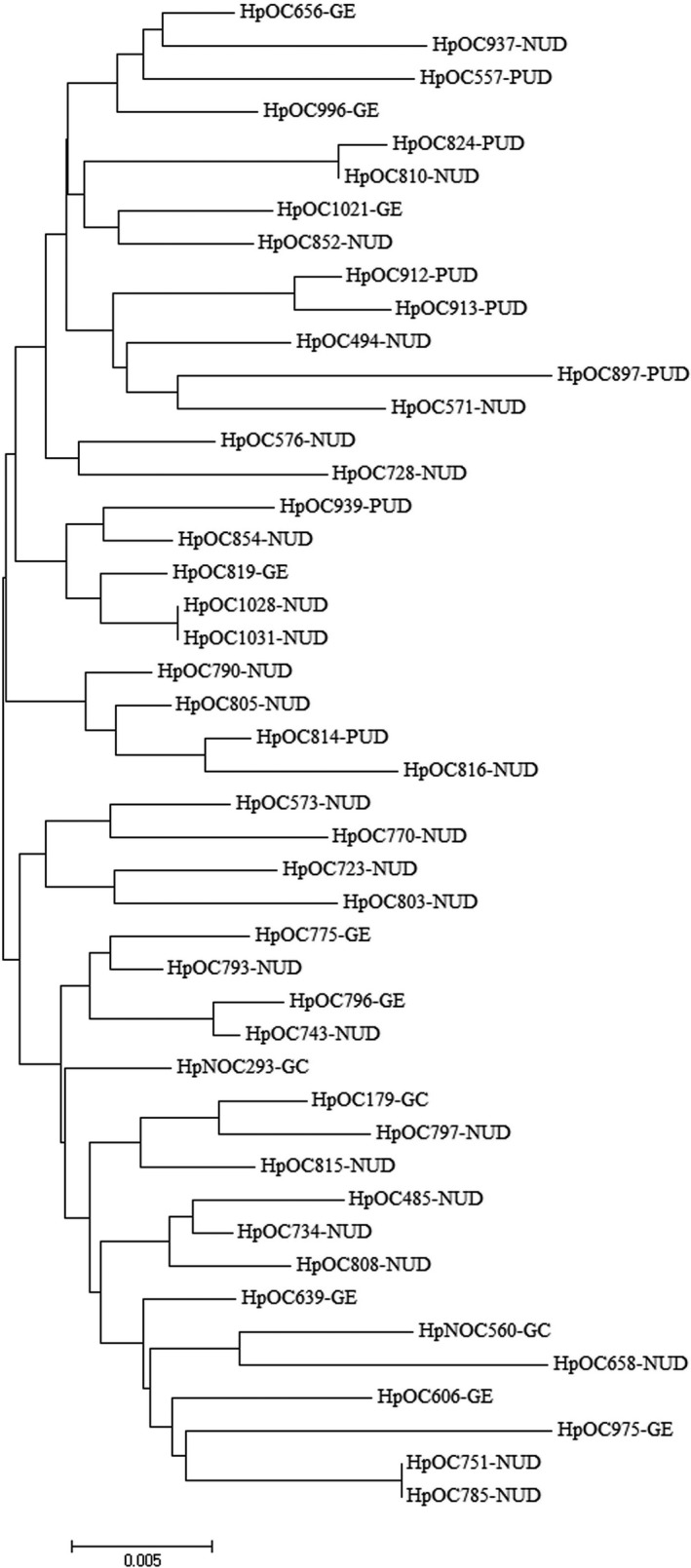
Phylogenetic tree of 46 *H pylori* clinical strains based on *cagL* nucleotide sequences. Neighbor‐joining tree of concatenated sequences was constructed using MEGA7 software with bootstrap method at 1000 replications. The evolutionary distances were computed using the Tamura 3‐parameter model

According to the nucleotide and amino acid sequence analysis, all of our *H pylori* strains expressed the RGD motif with no amino acid changes at residues 76‐78 (Figures 1 and 2). However, only a synonymous mutation (AG**A** to AG**G** transition) was detected in arginine residue of this tripeptide motif among 18/46 (39.1%) strains studied. Regarding the RHS motif, all of our strains also conserved the expression of FEANE pentapeptide motif with even no nucleotide polymorphisms at residues 86‐90. The C‐terminal SKIIVK (Ser‐Lys‐Ile‐Ile‐Val‐Lys) hexapeptide motif of CagL at residues 232‐237 was also conserved among all the strains in this study. However, three synonymous mutations were detected in serine (TC**G** to TC**A**), isoleucine (AT**C** to AT**T**), and valine (GT**C** to GT**T**) of this distal hexapeptide CagL motif among 46/46 (100%), 1/46 (2.2%), and 20/46 (43.5%) of the strains, respectively.

### 3.6 Association of CagLHM variants with disease outcomes and virulence genotypes

Based on the amino acid sequence comparison of CagLHM sequences, 10 motif variants were identified in the CagLHM located at residues 58‐62 in our studied strains (Table 3). Among these motifs, two common CagLHM sequences including NEIGQ and NKIGQ accounted for 21/46 (45.7%) and 9/46 (19.6%) of the sequences from the examined strains in this study. Moreover, most of the NUD (20/27, 74.1%), GE (6/9, 66.7%), and all of the GC (3/3, 100%) strains carried these two dominant motifs, whereas about half of the PUD strains (3/7, 42.8%) contained the NKMGK motif. Interestingly, a significant association was found between the isolates carrying NKMGK motif and PUD (*P* = 0.002). Three unique motifs for the CagLHM sequences including NKISQ (2/46, 4.3%), NKMGK (3/46, 6.5%), and DKMGQ (1/46, 2.2%) were identified among the translated CagL sequences in comparison with 508 sequences that were obtained from the previously deposited sequences in over‐mentioned databases.

**Table 3:** CagLHM sequence types and gastric disease associations of 46 *H pylori* isolates included in this study

CagLHM sequence	Total (n = 46)	Patient health status			
		NUD (n = 27)	PUD (n = 7)	GE (n = 9)	GC (n = 3)
NEIGQ	21	13	1	5	2
NKIGQ	9	7	—	1	1
DKIGQ	4	2	1	1	—
NKMGQ	3	2	—	1	—
NKMGK†	3	—	3	—	—
NKISQ†	2	2	—	—	—
NEIGK	1	—	1	—	—
DKMGE	1	1	—	—	—
DEIGQ	1	—	1	—	—
DKMGQ†	1	—	—	1	—
*P* Value*		0.153	**0.002**	0.735	1.0

CagLHM CagL hypervariable motif; GC, gastric cancer; GE, gastric erosion; NUD non‐ulcer dyspepsia; PUD peptic ulcer disease.

The “—” denotes none detected.

† Denotes that these CagLHM sequence types are novel and uniquely identified in our *H pylori* strains in comparison to global strains.

* *P* value indicates the significant difference between CagLHM diversity and different gastric diseases. The statistically significant relationships are calculated by two‐tailed Fisher's exact test and presented in bold.

Approximately half of the strains with *vacA* s1m2 allelic type (12/22, 54.5%) carried the NEIGQ, while about one‐third of the strains with *vacA* s1m1 genotype (5/14, 35.7%) contained this motif (Table 4). Additionally, most of the *babA2*‐positive strains (29/44, 65.9%) carried either NEIGQ or NKIGQ sequences. In contrast, nearly all of the *sabA*‐negative strains (7/8, 87.5%) had either NEIGQ or NKIGQ motifs in their CagLHM sequences. All *cagA*‐positive *H pylori* strains carrying ABC motif contained either one of the CagLHM variants identified, mostly NEIGQ (14/35, 40%) and NKIGQ (7/35, 20%) sequences. Among the strains with intact *cag*PAI structure, the most common CagLHM sequences were NEIGQ (14/33, 42.4%), NKIGQ (6/33, 18.2%), DKIGQ (4/33, 12.1%), NKMGK (3/33, 9.1%), and NKMGQ (2/33, 6.1%), respectively. All of the DKIGQ isolates carried an intact *cag*PAI. NEIGQ isolates having multiple C‐type EPIYA repeats and carrying intact *cag*PAI correlated with disease risk for PUD, GE, and GC (*P* = 0.021) than NUD. However, no significant association was found between other CagLHM sequences and virulence genotypes (*P* > 0.05).

**Table 4:** Differences in polymorphisms of various CagLHM sequence types of 46 *H pylori* isolates in relation to virulence genotypes and *cag*PAI integrity

Virulence genotypes	CagLHM sequence type polymorphism										
	NEIGQ (n = 21)	NKIGQ (n = 9)	DKIGQ (n = 4)	NKMGQ (n = 3)	NKMGK (n = 3)	NKISQ (n = 2)	NEIGK (n = 1)	DKMGE (n = 1)	DEIGQ (n = 1)	DKMGQ (n = 1)	Total (*n* = 46)
*vacA* s1m1	5	4	1	1	1	—	—	—	1	1	14
*vacA* s1m2	12	2	3	1	2	1	1	—	—	—	22
*vacA* s2m2	4	3	—	1	—	1	—	1	—	—	10
*babA2^+^*	20	9	4	3	3	1	1	1	1	1	44
*babA2^−^*	1	—	—	—	—	1	—	—	—	—	2
*sabA^+^*	16	7	3	3	3	2	1	1	1	1	38
*sabA^−^*	5	2	1	—	—	—	—	—	—	—	8
EPIYA motifs											
ABC	14	7	3	2	3	2	1	1	1	1	35
ABCC	4	1	1	—	—	—	—	—	—	—	6
ABCCC	1	—	—	—	—	—	—	—	—	—	1
Mixed type^a^	1	—	—	1	—	—	—	—	—	—	2
*cagA^−^*	1	1	—	—	—	—	—	—	—	—	2
*cag*PAI integrity											
Intact *cag*PAI	14	6	4	2	3	1	—	1	1	1	33
Partial *cag*PAI	7	3	—	1	—	1	1	—	—	—	13

CagLHM, CagL hypervariable motif; GC, gastric cancer; GE, gastric erosion; NUD, non‐ulcer dyspepsia; PUD peptic ulcer disease.

The “—” denotes none detected.

a Denotes multiple *cagA* EPIYA motifs of different sizes, indicating mixed infections.

### 3.7 Phylogenetic analysis of *cagL* gene

**Figure 3:**
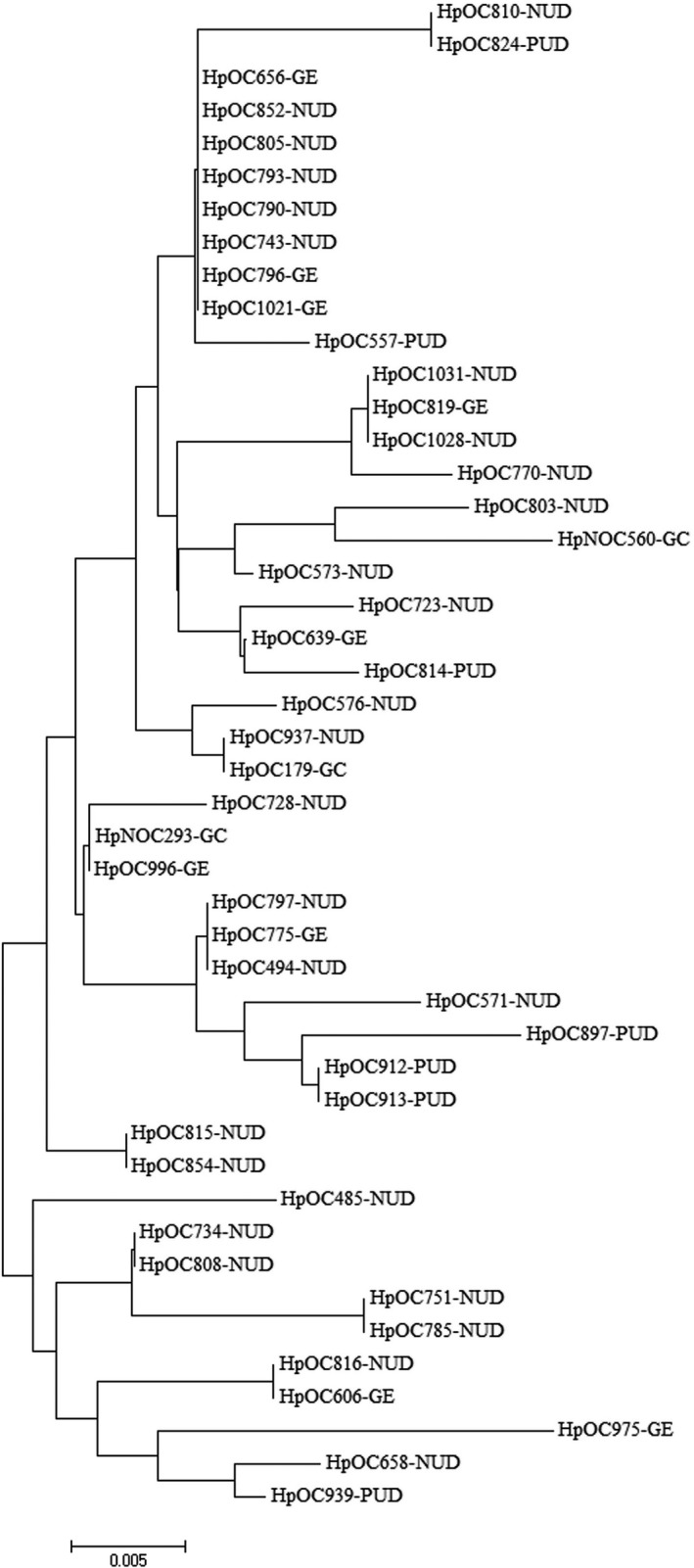
Phylogenetic tree of 46 *H pylori* clinical strains based on translated CagL amino acid sequences. Neighbor‐joining tree of concatenated sequences was constructed using MEGA7 software with bootstrap method at 1000 replications. The evolutionary distances were computed using the Poisson correction method

The edited DNA and amino acid sequences were aligned against reference sequence using ClustalW multiple alignment. The constructed Neighbor‐joining trees from *cagL* nucleotide and amino acid sequences of 46 *H pylori *isolates are presented in Figures 2 and 3, respectively. In general, no characteristic clusters were observed between DNA and amino acid sequences of CagL and different disease outcomes.

## 4 DISCUSSION

Functional *cag*PAI chromosomal DNA region, which is responsible for most of the *H pylori*‐related gastric pathologies and malignant phonotypes such as gastric atrophy and cancer, has been discovered in 1996.^14^, ^40^, ^41^ This most extensively studied part of the *H pylori* genome is present in approximately 95% of Asian isolates, whereas about 60% of Western isolates from low‐risk countries are *cag*PAI‐positive.^16^, ^36^, ^42^, ^43^ The *cag*PAI‐encoded T4SS is a multiprotein complex composed of homologs of *Agrobacterium tumefaciens *VirB/D proteins, which forms an extracellular pilus for injection of effector molecules into host target cells. CagL (HP0539), which was introduced as a putative VirB5 ortholog, is recruited to the surface of injection needle and binds to host cell β_1 _integrins via its surface‐exposed RGD motif essential for CagA translocation and the induction of IL‐8.^23^, ^44^, ^45^ Previous and some recent studies have shown that CagL is subject to genetic variations and positive or diversifying selection in some of its protein motifs that may affect its binding affinity to integrins.^14^, ^29^, ^30^, ^31^, ^32^, ^46^, ^47^ Some of these variations in amino acid sequences of CagL have been proposed to be involved in the cancer risk of infected patients.^29^, ^30^, ^31^, ^32^, ^33^, ^47^ Thus, in this study we determined the genetic variability in CagL on both nucleotide and amino acid sequence levels from *H pylori* strains isolated from patients with different clinical outcomes. We also examined the variations in *H pylori* CagLHM amino acid sequences in relation to other important virulence genotypes and different gastric diseases.

The results of the present study confirmed our previously published data indicating a very high frequency of*cagL* gene (97%) in Iranian *H pylori* strains, although in a non‐statistically significant relationship with clinical outcomes.^16^, ^34^ The high prevalence of *cagL* genotype in our study is in agreement with the results obtained from Malaysia and Singapore (>85%), Taiwan (98.6%), and India (86.6%).^31^, ^32^, ^48^ Moreover, in order to assess whether the CagL amino acid sequence polymorphisms and codon usages correlate with clinical outcomes, the *cagL* genes of 46 *H pylori* strains were sequenced. Our findings showed that amino acid residues at positions 41, 62, 122, and 171 had the greatest variability in their codon usages and were mostly non‐synonymous. The majority of the variations arose from nucleotide substitutions at either the first or second position of the putative progenitor codons. At position 41, we had V/T/A substitutions in different disease groups, in which V41 variant was predominantly observed in most of the strains from NUD (77.8%) and PUD (71.4%) and in all GE (100%) patients. This is in agreement with previous reports from Taiwan and Japan, where V41 variant found to be predominant in GC and non‐GC isolates.^32^, ^47^ By contrast, in our study V/T/A variants occurred equally in *H pylori* strains from GC patients. In addition, A41 variant was merely observed among patients with NUD (3/27) and GC (1/3). This finding is contrary to the study performed by Ogawa et al^47^ from Japan where A41 was only detected among CagL variants in GC isolates (2/10, 20%), although in a not significant relationship vs non‐GC isolates. In addition, the majority of our strains had Asparagine (N) at position 122, which is in line with the data obtained by Shukla et al^31^ from India where all of the strains were found to have N122 variant. In contrast, K122 was predominantly observed among Japanese and Taiwanese isolates.^32^, ^47^ However, no significant difference was seen between these amino acid variations and clinical outcomes (*P* > 0.05).

Our results revealed that N58 variant occurred at higher rate than D58 among studied *H pylori* strains, and more importantly, all of the strains from GC patients carried this amino acid variant in their CagL protein. By contrast, in previous studies from Taiwan, India, and Japan D58 substitution was more frequent than N58 in all disease groups.^31^, ^32^, ^47^ Additionally, none of the amino acid variants including Y58, G58, and M58 occurred in our CagL sequences, which had been previously reported from aforementioned studies. Similar to studies from Taiwan and Japan, the rate of E59 variant was higher in GC strains than non‐GC strains.^32^, ^47^ Conversely, Shukla et al^31^ found higher rate of K59 variant in CagL sequence of strains isolated from GC patients. Our results showed that N58E59 and N58K59 combined variants were more common in *H pylori *strains from GC patients. However, three different studies from Taiwan, India, and Japan reported a higher rate of the Y58E59, D58K59, and D58E59 amino acid combinations in GC patients, respectively.^31^, ^32^, ^47^ In another study in a Mexican patient cohort, 74 gene polymorphisms were observed in the *cagL*, which out of 24 analyzed variations, four showed a differential distribution between cases of cancer and gastritis (*P* < 0.05).^30^ Among these polymorphisms G166A (amino acidic change of A56 to T56) and A172G (amino acidic change of N58 to D58) were non‐synonymous, and two mutations including (A228G and C516T) were synonymous. Moreover, Yeh et al concluded that *H pylori* isolates carrying Y58E59 variant possibly exert stronger acid suppression during chronic infection and have strong binding affinity for integrin α_5_β_1_, which significantly promotes CagA translocation and phosphorylation as compared to wild‐type CagL.^32^, ^33^ However, their findings were found to be contradictory to those obtained by Tegtmeyer et al^49^ suggesting that Y58E59 mutation in CagL turned off the function of the T4SS for delivery of CagA into host cells.

Recently, analysis of CagL crystal structures revealed an elongated four‐helix bundle that seems to be evolutionarily unrelated to the proposed VirB5 orthologs.^45^, ^50^ Previous studies have proposed that the RGD tripeptide is located within a long α2 helix and is a critical motif in the structure of *H pylori* CagL pilus protein, able to bind and activate integrin α_5_β_1 _receptor on gastric epithelial cells.^22^, ^27^, ^45^, ^51^, ^52^ It has been also observed that RGD motif is at least partly involved in the cell signaling pathways leading to secretion of IL‐8, despite some controversy in the literature indicating that during infection mutation of the RGD motif in CagL does not affect the CagA translocation and IL‐8 induction.^22^, ^53^ Consistent with previous reports, all *H pylori* strains in the current study expressed the RGD motif in CagL sequences.^31^, ^32^, ^47^ These data highlight the importance of the RGD‐integrin interaction mediated translocation of CagA and also CagL‐dependent signaling pathway for induction of proinflammatory cytokines such as IL‐8. In addition to RGD motif, CagL protein contains another motif named RHS pentapeptide or FEANE in spatial proximity to the RGD sequence, which is proposed to enhance the CagL interactions with β_1_ integrins.^24^ Notably, the RHS and the C‐terminal SKIIVK hexapeptide motifs were universally expressed in all of our strains, which is partly in line with previous reports from Taiwan and Japan.^32^, ^47^ As previously suggested, these findings underscore the significance and essential roles of aforementioned motifs especially the highly conserved SKIIVK sequence that is present nearly identical in other *cag*PAI components such as CagI and CagH, in the stability, subcellular transport of CagL, pilus formation, and biogenesis, and subsequently in the CagA translocation and IL‐8 induction from epithelial cells.^54^

Based on previous studies, certain variants of CagLHM sequence containing five hypervariable amino acid residues (58, 59, 60, 61, and 62), which is located upstream of the RGD motif have been associated with gastric carcinogenesis.^28^, ^31^, ^32^, ^33^, ^47^ Recently, a global analysis of geographical diversity and polymorphism was carried out within the CagLHM motif of more than 500 amino acid sequences of CagL in gastric cancer‐associated *H pylori* isolates worldwide.^29^ Accordingly, 33 CagLHM sequence combinations with diverse geographical prevalence have been identified in different regions of the world particularly among Asian countries showing extensive diversity with 20 out of 33 (60.6%) unique CagLHM motifs in this region. Additionally, four motifs including DKMGE, NEIGQ, NKIGQ, and DKIGK were identified as the most common CagLHM sequences and accounted for >75% of available sequences from *H pylori* strains worldwide. Interestingly, we detected 10 variants of CagLHM motif within the 46 CagL sequences revealing substantial diversity in Iranian strains. We also identified two common motifs including NEIGQ and NKIGQ accounted for 45.7% and 19.6% of the sequences, respectively, which is in agreement with data from European and West/Central/South Asian countries where these two motifs were predominant.^29^ These data once again highlight that Iranian *H pylori *strains shared ancestral origins with the European counterparts and were intermingled with strains assigned to the hpEurope population.^55^, ^56^ Our results showed that all of the strains isolated from GC patients carried one of the two over‐mentioned predominant motifs. We also found a strong association between the NKMGK CagLHM and PUD (*P* = 0.002) clinical outcome, where NKMGK was detected as dominant motif and none the isolates from other clinical outcomes contained this type of motif. More importantly, three unique motifs including NKMGK, NKISQ, and DKMGQ accounted for 6.5%, 4.3%, and 2.2% of the sequences, respectively, were identified among our strains that were not reported previously. In addition, in this study and for the first time we examined the co‐occurrence of specific CagLHM motifs with the main virulence‐associated genes of *H pylori*. Notably, our results revealed that the NEIGQ isolates with multiple C‐type EPIYA repeats that carried intact *cag*PAI correlated with increased disease risk for PUD, GE, and GC (*P* = 0.021) than NUD. This may indicate the critical importance of this CagLHM motif in the pathogenesis of *H pylori *strains in a synergistic relation to other virulence genotypes. Moreover, further studies are required to investigate the possible influence of specific CagLHM motifs on the CagL interaction with host cell integrins and also their impacts on expression and function of other components of *H pylori *T4SS.

## 5 CONCLUSIONS

In conclusion, we identified putative novel variants of CagL sequences especially in the CagLHM motif, which may have crucial effect on the activity and function of T4SS and its pilus formation. Among the ten different CagLHM variants identified, all of the DKIGQ isolates carried an intact *cag*PAI that may be concluded that this motif was mostly overrepresented by the hypervirulent strains, albeit not statistically significant. Moreover, our findings demonstrated that the majority of Iranian *H pylori* strains were *cagL* positive, and all such strains expressed the RGD motif, the so‐called RHS or FEANE pentapeptide and the C‐terminal SKIIVK motif within their CagL protein. The very high‐level genetic conservation seen in these sequences, possibly due to different evolutionary selection pressures, underscores the importance of aforementioned motifs in the RGD‐dependent and RGD‐accessory integrin binding of CagL protein, as well as in protein‐protein interaction with other T4SS components to facilitate the CagA injection into host cells. Taken together, more studies using a large number of *H pylori* strains from patients with different disease outcome are necessary to further define the function of specific CagL amino acid polymorphisms especially the novel CagLHM variants in certain intracellular signaling pathways and subsequently with respect to disease progression and clinical outcome.

## CONFLICT OF INTEREST

The authors declare that they have no conflict of interest.
